# Mix Design and Performance Analysis of Concrete with Limestone Powder Admixture

**DOI:** 10.3390/ma19112348

**Published:** 2026-06-02

**Authors:** Fangyuan Gong, Mingyuan Xu, Jinbiao Wu, Penghua An, Weihao Shi

**Affiliations:** 1 School of Civil and Transportation Engineering, Hebei University of Technology, 5340 Xiping Road, Beichen District, Tianjin 300401, China; 202331605019@stu.hebut.edu.cn; 2 Huitong Construction Group Co., Ltd., No. 69, Shiji East Road, Baoding 074000, China

**Keywords:** concrete, limestone powder, mechanical performance, durability, response surface method (RSM), digital image correlation (DIC)

## Abstract

The use of waste limestone powder from mining to replace natural sand in concrete improves its mechanical performance and durability, reduces production costs, and benefits the ecological environment. In the initial mix design, limestone powder was substituted for natural sand as fine aggregate at specific ratios under constant total mass, investigating the influence of water-to-binder ratio and limestone powder replacement rate on concrete strength to establish preliminary value ranges for the optimal parameters. The secondary mix design employed Design Expert software 13 to develop a response surface model, analyzing the impact of water-to-binder ratio and limestone powder replacement rate on key mechanical properties like compressive and flexural strength, as well as the durability of the concrete containing limestone powder. Furthermore, Digital Image Correlation (DIC) was utilized to monitor the stress–strain state on the concrete surface, with the resulting strain cloud maps used to characterize crack initiation locations and propagation patterns. Overall, the results indicate that the observed performance evolution was governed by the coupled influence of limestone powder replacement rate and total powder content. Under the present experimental conditions, limestone powder demonstrated potential as a sustainable fine aggregate substitute; however, its intrinsic contribution could not be completely isolated because of the simultaneous variation in powder content. The study further clarifies the coupled influence of limestone powder replacement rate and total powder content, while integrating response surface optimization and DIC analysis method to provide a practical framework for limestone powder concrete design and engineering application.

## 1. Introduction

With industrialization and economic development, the demand for building materials continues to grow. Concrete, being the most extensively utilized construction material worldwide, has a key constituent, cement, with an annual output already approximating 4.0 billion tons [1], and this figure is forecasted to rise to 5.8 billion tons by 2050 [2]. Aggregates constitute approximately 70% of concrete volume, with an annual consumption of about 8–12 billion tons. Aggregates can be classified by particle size into coarse aggregates (size greater than 4.75 mm) and fine aggregates (less than and equal to 4.75 mm, i.e., sand). Sand plays a critical role in concrete, not only as a primary component but also significantly influencing the workability, strength, durability, shrinkage, and bulk density. As a filling material, sand effectively fills pores to enhance strength, reduces volume changes during hydration, and increases load-bearing capacity by augmenting particle count, offering advantages over pure cement paste. Nevertheless, natural aggregates, especially river sand, a fine aggregate renowned for its high performance, are facing growing scarcity and declining quality. Over-exploitation has caused a series of environmental issues, including soil erosion, river ecosystem damage, and bank instability, while also driving up costs. Consequently, identifying sustainable substitutes for natural sand has emerged as a pressing requirement for the industry. Manufactured sand has therefore emerged as a viable substitute and is now widely used. Nevertheless, during its production process, a significant amount of stone powder is inevitably generated, typically accounting for 10–15% of the total, and in some cases exceeding 20%, which is far beyond the limits specified by existing standards. To comply with these requirements, excess stone powder is often removed through water washing or air separation. However, these processes not only increase production complexity and cost but also lead to the loss of fine particles (0.15–0.60 mm), disrupting the original particle size distribution. Moreover, such treatments result in the waste of mineral and water resources and may cause additional environmental pollution [3]. Given that manufactured sand accounts for approximately 30% of the total sand and gravel production, and considering that the annual output in China has reached about 20 billion tons in recent years, the amount of stone powder generated is estimated to be as high as 600 million to 1.2 billion tons per year [4]. The disposal and utilization of this large quantity of stone powder have therefore become critical environmental and engineering challenges. In recent years, the resource utilization of solid waste has attracted increasing global attention under the framework of sustainable development and the circular economy. Various industrial by-products and construction wastes, such as fly ash, slag, and stone powder, are being explored as alternative materials in construction. Among these, stone powder exhibits considerable potential due to its fine particle size and mineralogical characteristics. Its effective incorporation into concrete or other building materials could not only mitigate environmental impacts but also reduce dependence on natural resources. Therefore, the efficient utilization of stone powder as a solid waste resource has become an urgent and important research topic in the field of construction materials.

Due to the wide variety of raw materials used in manufactured sand production, the physical and chemical properties of the resulting stone powder can vary significantly, leading to different effects on concrete performance. Among the various types of stone powder, limestone powder has attracted considerable attention in recent years because of its wide availability, low cost, and large-scale production capacity [5]. The incorporation of limestone powder into concrete not only reduces material costs but also contributes to lowering carbon dioxide emissions, thereby promoting the development of green and sustainable construction materials [6,7,8].

Currently, limestone powder can be incorporated into concrete through two primary approaches: (1) as a partial replacement for fine aggregates, utilizing its filling and packing effects [9,10], and (2) as a partial replacement for cementitious materials [11,12]. Its fine particles can provide nucleation sites and improve microstructure; however, excessive content may lead to a dilution effect. In addition, due to the irregular and rough surface texture of limestone powder, high dosages tend to increase water demand, thereby reducing the workability of concrete [13]. Previous studies have reported that an appropriate amount of limestone powder can enhance concrete performance. For example, Wang et al. found that as the limestone powder content increased from 0% to 20%, the microstructure of both the interfacial transition zone and the cement paste first became denser and then deteriorated, with optimal compressive strength achieved at approximately 10% replacement [14]. Similarly, Islam et al. demonstrated that a 10% replacement level provided the best balance between mechanical properties and durability due to the combined nucleation and filling effects, while avoiding the adverse dilution effect at higher dosages [15]. In addition to conventional concrete, limestone powder has also been widely studied in high-strength and ultra-high-performance concrete (UHPC). For instance, Abellán-García et al. incorporated limestone powder as a partial replacement for cement and silica fume in UHPC and reported compressive strengths exceeding 150 MPa at 90 days, along with improved durability performance [16]. Furthermore, statistical and modeling approaches have been proposed to optimize limestone powder dosage in high-strength concrete systems [17]. However, the influence of limestone powder on durability remains complex and sometimes contradictory. Li et al. reported that low limestone powder content (< 8 wt%) can refine the microstructure and reduce chloride ion diffusion, whereas higher contents may have adverse effects [18]. Similarly, Pham et al. observed that replacing 5% of fine aggregate with limestone powder reduced sulfate resistance, indicating potential durability concerns under certain conditions [19].

Recent studies have further demonstrated that the performance evolution of limestone powder concrete cannot be interpreted solely from the perspective of replacement ratio, but should also be considered in relation to particle packing characteristics and microstructural development. Particle packing theory has increasingly been employed to explain the improvement in mechanical properties and durability of cement-based materials. It has been reported that optimized particle size distribution can effectively enhance packing density, refine pore structure, and reduce chloride diffusivity by improving the compactness of the matrix. In particular, synergistic optimization of aggregate grading and cementitious particle distribution was found to significantly improve resistance to chloride transport through dual-scale packing mechanisms [20]. From the durability perspective, chloride transport in concrete has been recognized as a complex coupled process involving pore structure, chloride binding capacity, tortuosity, and moisture transport. Recent studies have shown that the incorporation of limestone powder may alter both pore characteristics and chloride binding behavior, thereby affecting chloride diffusion mechanisms. The reduction in chloride transport has been attributed not only to pore refinement but also to changes in the chemical binding process and transport pathways [21,22,23]. Therefore, the evaluation of chloride resistance should be interpreted together with microstructural evolution and packing characteristics. In recent years, Digital Image Correlation (DIC) has been increasingly applied in cement-based materials because of its capability to provide full-field strain information and visualize crack evolution processes. Compared with conventional displacement measurements, DIC enables the identification of strain localization, crack initiation, and failure development, providing a more comprehensive understanding of deformation mechanisms in concrete materials [24]. Nevertheless, the application of DIC in limestone powder concrete remains relatively limited. Furthermore, optimization approaches have been widely introduced in concrete design to establish relationships between mixture parameters and engineering performance. Statistical methods such as response surface methodology (RSM) have been demonstrated to effectively evaluate interactions among variables and optimize multi-objective performance indices. However, most existing optimization studies concerning limestone powder concrete have focused on strength prediction, while relatively limited attention has been paid to the combined optimization of mechanical properties, durability, and deformation behavior [16].

Although extensive research has been conducted on the incorporation of limestone powder in concrete, most existing studies have primarily focused on its use as a supplementary cementitious material or cement replacement. In contrast, comparatively limited attention has been paid to the direct substitution of fine aggregate by limestone powder, particularly at high replacement levels. In practical applications, the utilization of quarry-derived limestone powder as a sand substitute is of considerable engineering significance because it allows direct consumption of waste materials while reducing dependence on natural river sand. Furthermore, in most previous investigations, the influence of limestone powder dosage has generally been interpreted as an independent material effect. However, when limestone powder is used to replace fine aggregate, the total powder content of the concrete system increases simultaneously with the replacement rate. Consequently, the effects of limestone powder replacement and total powder content become inherently coupled. This coupling relationship has rarely been systematically discussed in previous studies, although it may substantially affect particle packing characteristics, pore structure, mechanical performance, and durability. In addition, previous studies have mainly focused on conventional performance evaluation, whereas limited research has combined statistical optimization methods with full-field deformation characterization techniques to investigate limestone powder concrete systems. The crack evolution mechanism and strain localization behavior under different mixture conditions remain insufficiently understood.

Therefore, the novelty and contribution of this study can be summarized as follows: (1) Limestone powder was employed as a direct substitute for fine aggregate rather than as a cementitious replacement material, and its feasibility at high replacement levels was systematically evaluated. (2) The coupled influence of limestone powder replacement rate and total powder content was analyzed, and the governing role of powder content in concrete performance was clarified. (3) Response surface methodology (RSM) and digital image correlation (DIC) were jointly introduced to optimize mix proportion and characterize crack evolution behavior. (4) A practical mix design framework considering mechanical properties, durability, workability, and engineering applicability was proposed.

## 2. Materials and Methods

### 2.1. Selection of Experimental Materials

The cement employed in this research was P·O 42.5 grade supplied by Hebei Jinyu Dingxin Cement Co., Ltd., Baoding, China, with a specific surface area of 380 m^2^/kg. Coarse aggregate was crushed stone produced by Baoding Xuding Road Engineering Co., Ltd., Baoding, China, consisting of 5–10 mm, 10–20 mm, and 16–31.5 mm particles blended at 17%:70%:13% to form a continuous 5–31.5 mm gradation; the apparent density of the coarse aggregate was 2822 kg/m^3^. Fine aggregate was natural sand meeting Zone II medium sand specifications, with an apparent density of 2683 kg/m^3^. Slag powder was obtained from Xibaipo New Energy Co., Ltd., Shijiazhuang, China, classified as Grade S95, with a specific surface area of 424 m^2^/kg. Fly ash was Class F Grade I fly ash from Huaneng International Power Co., Ltd., Dezhou, China Dezhou Power Plant, with a fineness of 22%. Ordinary tap water served as mixing water, while a polycarboxylate-based high-range water reducer was used as the chemical admixture. The stone powder, mainly comprising limestone powder, was derived from quarry waste materials. The basic indicators are shown in Table 1. Its chemical composition, summarized in Table 2, was provided by the supplier and verified to comply with the specifications outlined in Chinese national standards on Limestone Powder Used for Cement, Mortar and Concrete (GB-T 35164-2017) [25]. The material is characterized by a high CaCO_3_ content, indicating that it can be classified as a typical limestone powder.

**Figure 1:**
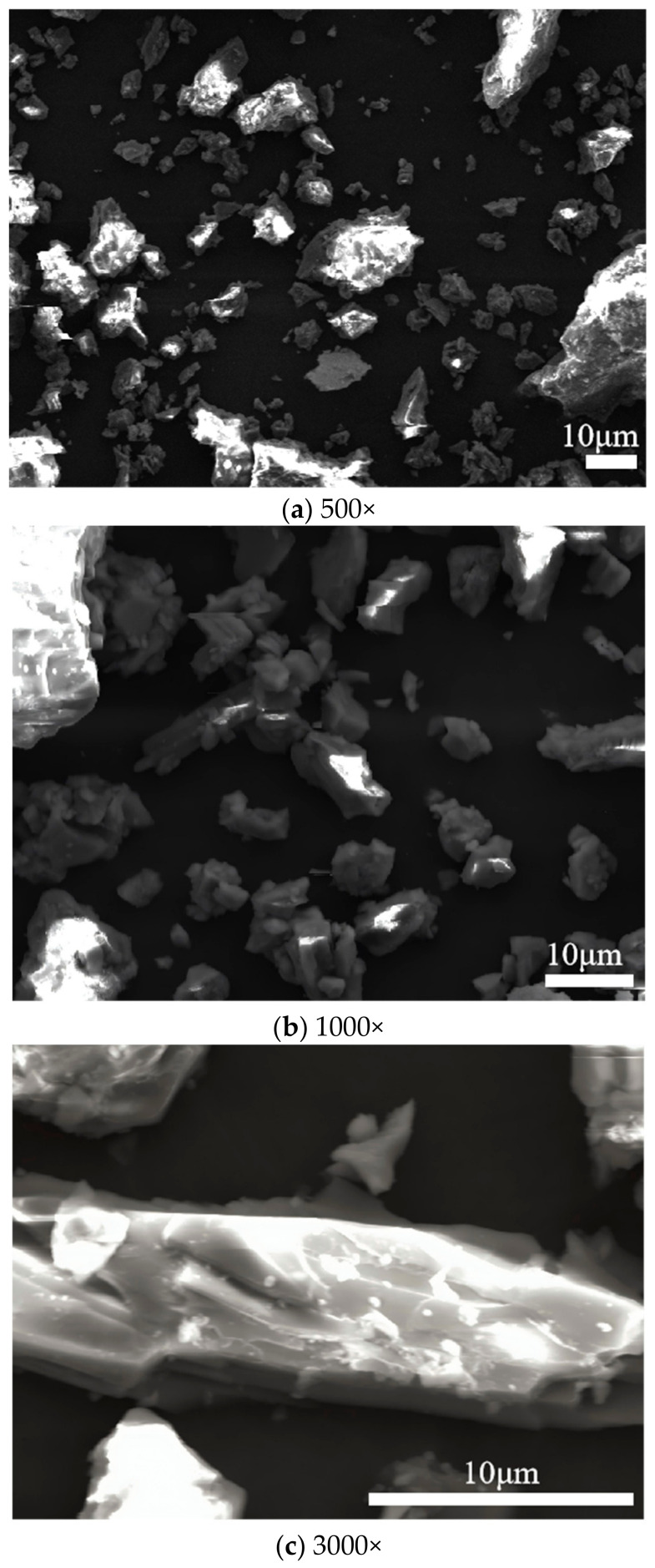
SEM images of limestone powder at different magnifications.

Figure 1a–c present Scanning Electron Microscopy (SEM) JSM-7800F, JEOL Ltd., Tokyo, Japan, images of the limestone powder at magnification levels of 500×, 1000×, and 3000× respectively. As shown in Figure 1, unlike the spherical fly ash particles, this limestone powder exhibits angular morphology and rough surface texture. The angular shape may indicate higher specific surface area at identical particle sizes, consequently requiring more water for surface wetting [26].

### 2.2. Aggregate Gradation Design

**Figure 2:**
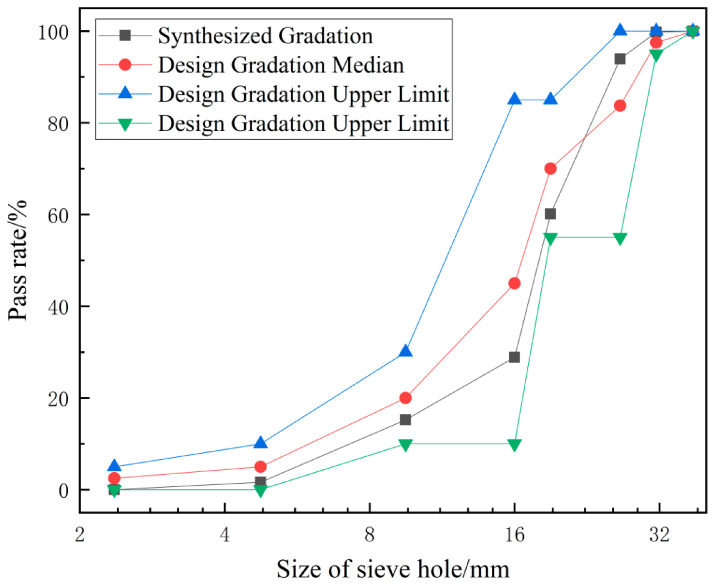
Design grading curve.

Based on the Specification for Mix Proportion Design of Ordinary Concrete (JGJ55-2011) [27], the grading design for the coarse aggregates to achieve a continuous grading from 5 mm to 31.5 mm was designed in this study. The design gradation table is shown in Table 3 and its curves are plotted in Figure 2.

### 2.3. Mix Proportion Design

The mix proportions were determined in accordance with relevant design specifications and standard calculation procedures for concrete. The water–binder ratio, binder content, and aggregate proportions were selected to meet the target strength grade and workability requirements. Limestone powder was introduced as a partial replacement for fine aggregate at different replacement levels. All mixtures were prepared following the same design framework to ensure comparability The material quantities are shown in Table 4. It should be noted that no optimization procedure was involved at this stage. The mix design served as the basis for subsequent experimental investigation and modeling. Three water-to-binder ratios of 0.38, 0.43, and 0.48 were adopted. Limestone powder was used to replace natural sand at rates of 0%, 20%, 40%, 60%, 80%, and 100%, in order to evaluate its suitability as a fine aggregate substitute in concrete. The workability of all mixtures was assessed using the slump test. To ensure comparable fresh properties, the dosage of superplasticizer was adjusted so that the slump values of all mixtures remained within a narrow range of 200–220 mm. This approach minimized the influence of workability variations on the experimental results. All specimens were cast and compacted using an identical vibration procedure to reduce variability associated with compaction. The small variation in slump values indicates that the mixtures exhibited similar plasticity; therefore, the influence of fresh-state properties on the measured mechanical performance can be considered negligible.

Since the use of limestone powder replaces the sand, it will increase the content of powders in the concrete. Therefore, the content of powders needs to be calculated (powder = limestone powder + cementitious material). The powder content is shown in Table 5.

### 2.4. Performance Testing

#### 2.4.1. Mechanical Properties Test

**Figure 3:**
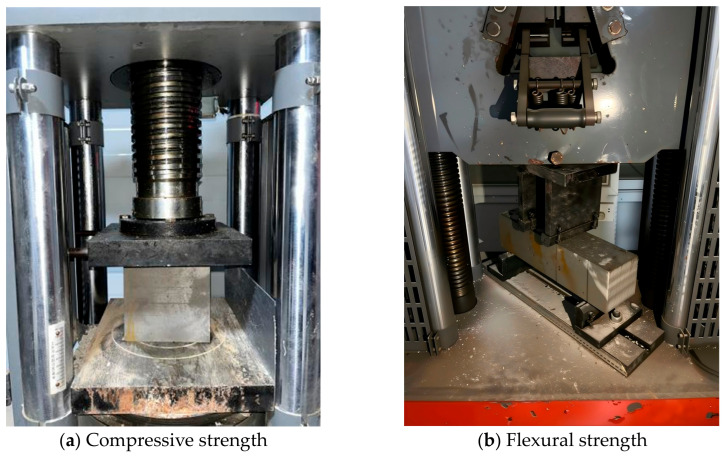
Tests of mechanical properties of concrete mixed with limestone powder.

According to the Test Methods of Cement and Concrete for Highway Engineering (JTG 3420-2020) [28], the cube compressive strength specimens have a side length of 150 mm; the prism flexural strength specimens dimensions are 600 mm × 150 mm × 150 mm (length × width × height). The strength testing for limestone powder concrete is shown in Figure 3.

#### 2.4.2. Durability Test

The importance of durability in cement concrete cannot be underestimated, as it directly impacts the safety, service lifetime, economic value, sustainability, and societal benefits of concrete structures, constituting a fundamental performance parameter of equal importance to mechanical strength. A crucial direction in modern concrete technology development is to significantly enhance concrete durability while maintaining strength, thereby achieving structures with long service life, low maintenance, and sustainability. Therefore, chloride ion penetration resistance tests are conducted on cement concrete to evaluate its resistance to chloride ingress and prevent the detrimental effects of steel reinforcement corrosion.

**Figure 4:**
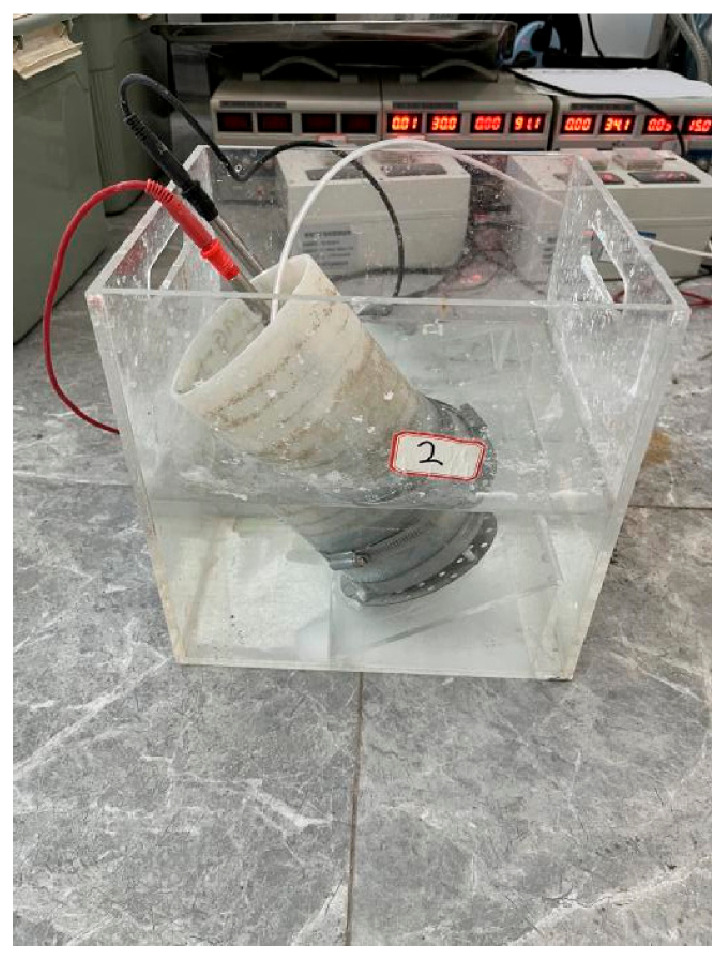
Test site of resistance to chloride ion penetration of concrete mixed with limestone powder.

According to the Test Methods of Cement and Concrete for Highway Engineering (JTG 3420-2020) [28], the rapid chloride migration (RCM) method was employed to determine the coefficient of non-steady-state chloride migration in concrete. Pre-prepared specimens were placed in rubber sleeves and positioned in test cells. The TR-RCM-6 chloride diffusion coefficient tester was used to apply electric current, and data were recorded following standardized procedures. After testing, the power was turned off and specimens were removed, then split axially into two halves. A 0.1 mol/L AgNO_3_ solution was sprayed as a colorimetric indicator on the split surface. After standing for 15 min, the distance from the color front to the bottom surface of the specimen was measured. The test setup for chloride penetration resistance of limestone powder concrete is shown in Figure 4. The calculation formula for the non-steady-state chloride migration coefficient of cement concrete is

(1) $$D_{RCM}=\frac{0.0239\times \left(273+T\right)L}{\left(U-2\right)t}\left[X_{d}-0.0238\sqrt{\frac{\left(273+T\right)LX_{d}}{U-2}}\right]$$

where $D_{RCM}$ represents the non-steady-state chloride migration coefficient of cement concrete (m^2^/s); U is the absolute value of the applied voltage (V); T is the average value of the initial and final temperatures of the anolyte solution (°C); L is the specimen thickness (mm), accurate to 0.1 mm; $X_{d}$ is the average chloride penetration depth (mm), accurate to 0.1 mm; t is the test duration (h).

#### 2.4.3. Workability Test

The slump test was conducted in accordance with the Test Methods of Cement and Concrete for Highway Engineering (JTG 3420-2020) [28]. A standard slump cone (height: 300 mm; top diameter: 100 mm; bottom diameter: 200 mm) and a steel tamping rod (diameter: 16 mm; length: 600 mm) were used.

The concrete mixture was placed into the slump cone in three approximately equal layers. Each layer was compacted by 25 strokes of the tamping rod, applied uniformly in a spiral pattern from the edge toward the center, in accordance with the standard procedure. After the top layer was compacted, the surface was leveled, and any excess concrete around the base of the cone was carefully removed.

The cone was then lifted vertically in a steady motion within 3–7 s. The slump value was determined by measuring the difference between the height of the cone and the highest point of the slumped concrete using a steel ruler, with a measurement accuracy of 1 mm.

**Figure 5:**
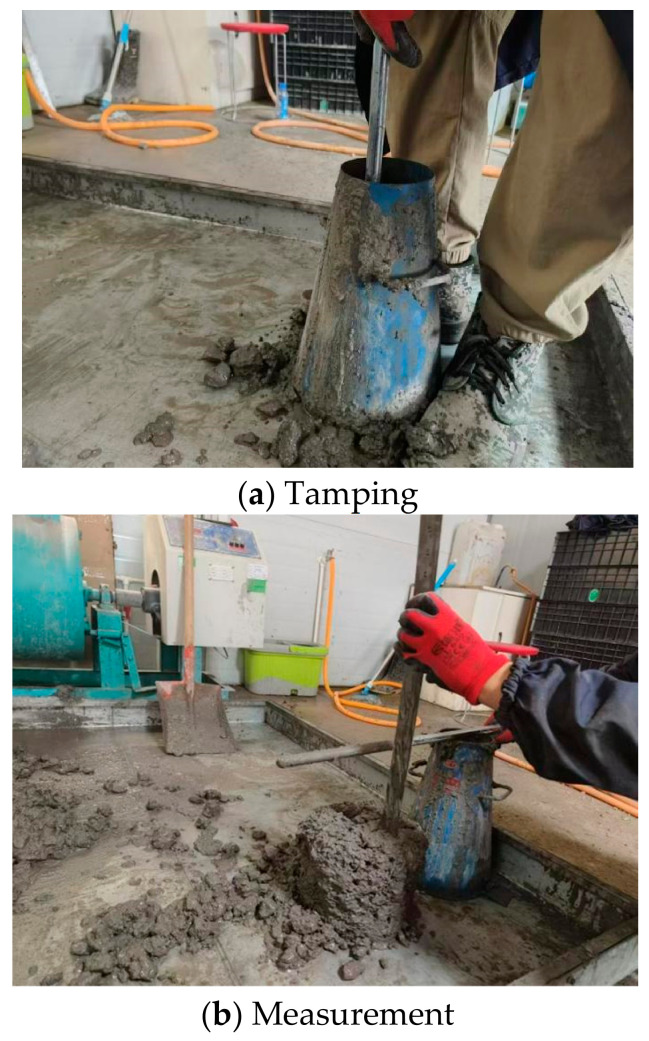
Slump detection process.

No mechanical vibration was applied during the test, and all procedures were carried out under standard laboratory conditions. The slump testing procedure is illustrated in Figure 5. To ensure the reliability of the measurements, the slump test was repeated for each mixture, and consistent results were obtained within a narrow variation range. The reported values represent the measured results rather than visual interpretation of the slump shape. All procedures were conducted under controlled laboratory conditions in accordance with the standard specifications.

### 2.5. Statistical Analysis

For each mixture group, three specimens were prepared and tested for mechanical and durability evaluation. The reported values were expressed as the mean values of three measurements, while the standard deviation (SD) was used to characterize the experimental variability. To further evaluate the robustness of the experimental results, 95% confidence intervals (CI) were estimated according to Student’s t-distribution because of the limited sample size (*n* = 3). Details can be found in the Supplementary Materials. The confidence interval was calculated as

(2) $$CI=\bar{x}\pm t_{n}\frac{SD}{\sqrt{n}}$$

where $\bar{x}$ is the mean value, SD is the standard deviation, *n* is the number of specimens, and t is the critical value corresponding to the 95% confidence level. The uncertainty of the measurements was assessed through the statistical dispersion of repeated tests and is represented by error bars in the figures.

## 3. Results and Analysis

### 3.1. Mechanical Properties

#### 3.1.1. Compressive Strength Analysis

**Figure 6:**
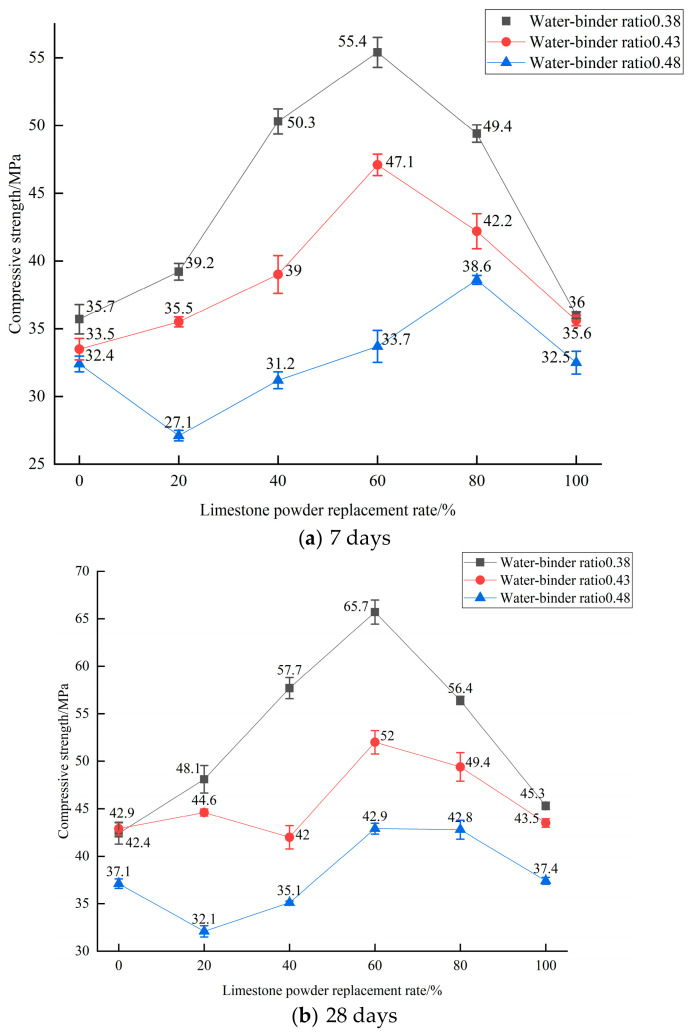
Influence of limestone powder replacement rate on the compressive strength of concrete mixed with limestone powder at different curing ages.

**Figure 7:**
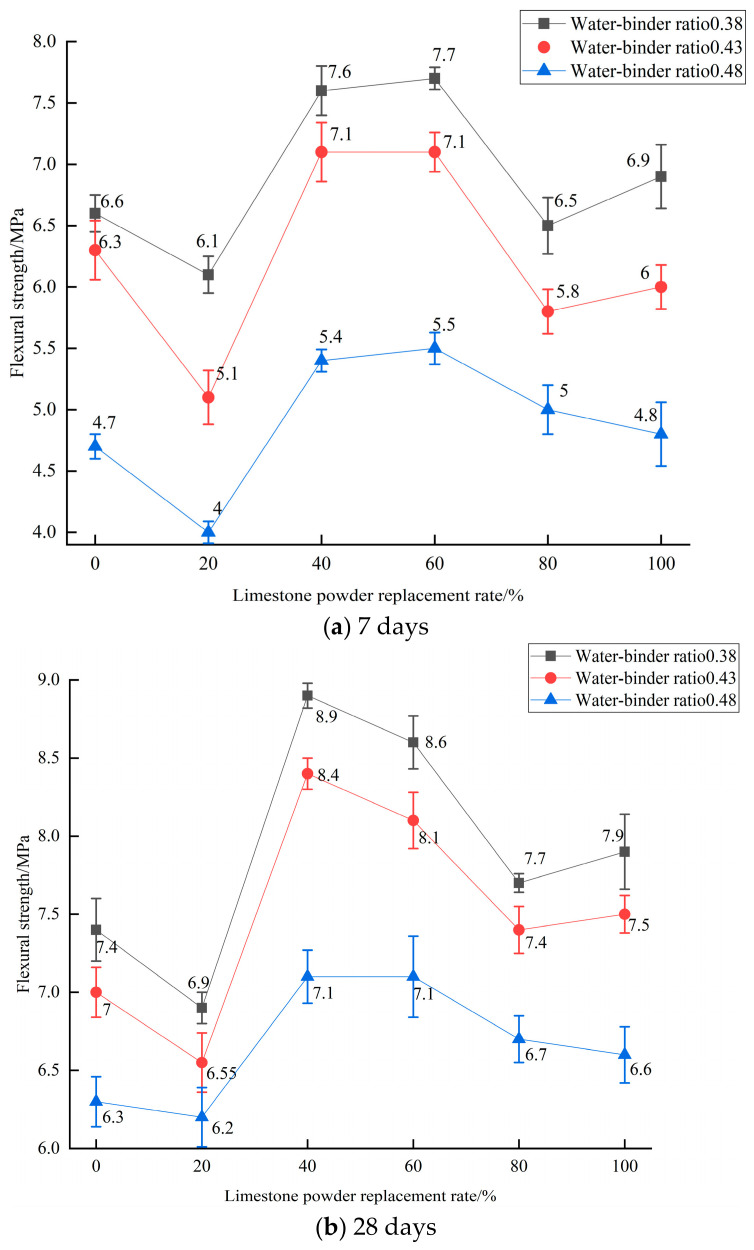
Influence of limestone powder replacement rate on the flexural strength of concrete mixed with limestone powder at different curing ages.

**Figure 8:**
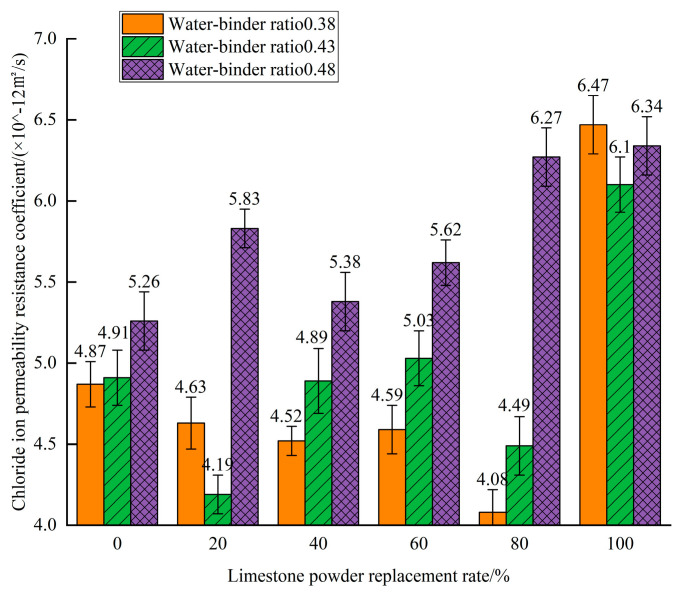
Effect of limestone powder substitution rate on chloride ion permeability resistance of concrete mixed with limestone powder at different water–binder ratios.

For each mixture group, three specimens were prepared and tested. The reported values were obtained as the average values of three measurements, and the standard deviation was used to characterize the experimental variability. Error bars presented in the figures represent the statistical dispersion of the results. The results presented in Figure 6, Figure 7 and Figure 8 correspond to the mean values of three specimens, and error bars are included to indicate the experimental variability. The compressive strength exhibited an increasing–decreasing trend with increasing limestone powder replacement rate. The optimum performance was generally observed at intermediate replacement levels. At lower replacement levels, the increase in powder content may contribute to improved internal compactness. However, excessive replacement resulted in performance deterioration, which could be associated with disruption of the aggregate framework and excessive accumulation of fines. Wang et al. reported that limestone powder mainly affects cement-based materials through filler, nucleation, dilution, and chemical effects, and may accelerate early hydration by promoting C–S–H formation [29]. Recent studies indicated that fine limestone particles may provide nucleation sites and influence hydration kinetics depending on particle size and dosage [30].

It should be noted that the observed behavior reflected the coupled influence of replacement rate and total powder content rather than the isolated material effect of limestone powder.

Overall, the observed compressive strength variation should be interpreted as a coupled effect associated with simultaneous changes in limestone powder replacement rate and total powder content. Under the present experimental conditions, the influence of powder content appears to play a dominant role, while the intrinsic effect of limestone powder cannot be independently quantified.

**Figure 9:**
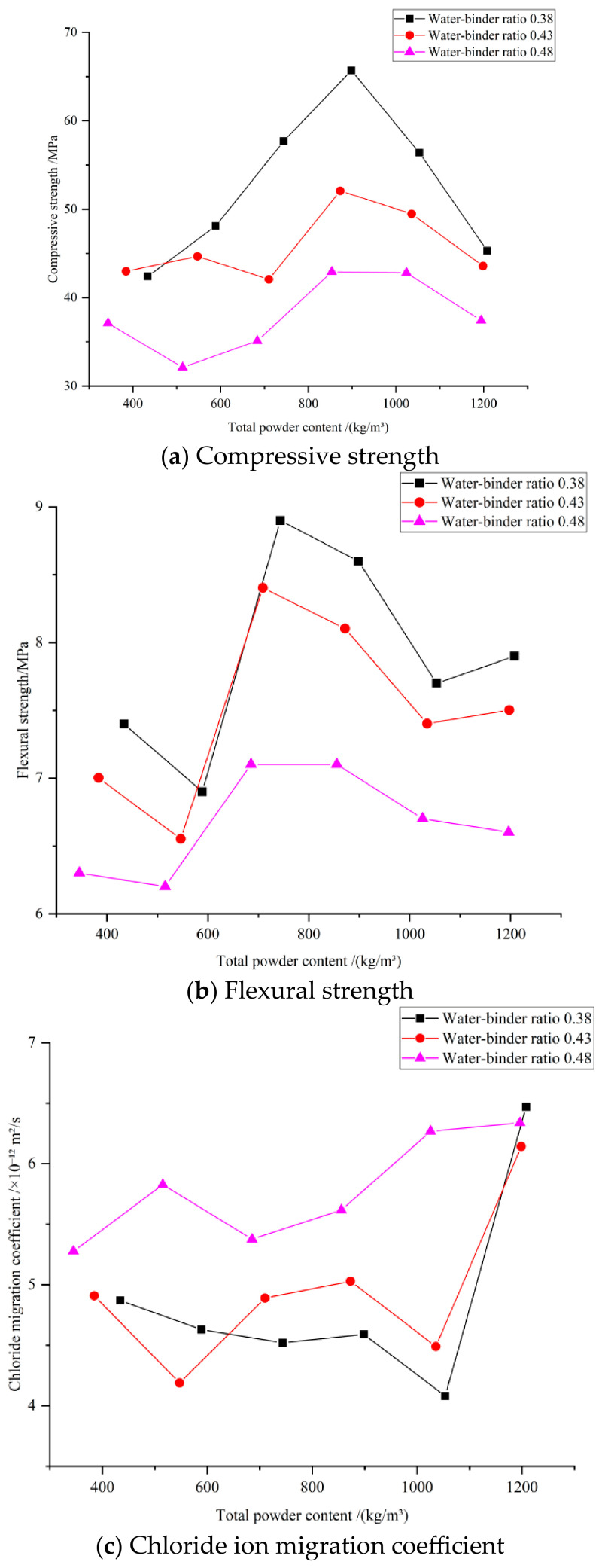
Relationships between total powder content, compressive strength, flexural strength, and chloride ion migration coefficient under different water–binder ratios.

To further elucidate the coupled effect between limestone powder replacement rate and total powder content, the experimental results were replotted as a function of total powder content, as shown in Figure 9. It can be observed that both compressive strength and flexural strength exhibit a clear dependence on total powder content, with peak values occurring at intermediate levels (approximately 800–900 kg/m^3^). Similarly, the chloride ion migration coefficient reaches a minimum within this range. This consistent trend across different performance indicators suggests that the observed enhancement is mainly governed by the increase in total powder content and the associated improvement in particle packing density. Therefore, under the present experimental conditions, total powder content appears to play a dominant role, while the intrinsic effect of limestone powder cannot be fully isolated.

#### 3.1.2. Flexural Strength Analysis

The variation in flexural strength generally followed the trend observed for compressive strength, indicating similar governing mechanisms. The optimum performance was obtained at intermediate replacement levels, whereas excessive replacement adversely affected the load transfer capability of the internal framework.

Therefore, it can be inferred that the variation in flexural strength is primarily governed by the combined influence. The present results demonstrate that the variation in flexural strength was associated with coupled changes in powder content and replacement rate. Consequently, the intrinsic effect of limestone powder could not be independently evaluated.

To address the coupling effect between limestone powder replacement and total powder content, the experimental results were further analyzed by considering total powder content as an independent variable. It was observed that both compressive strength and durability exhibited a strong dependence on total powder content, with optimal performance corresponding to intermediate powder levels, approximately 800–900 kg/m^3^, regardless of the specific replacement ratio. This suggests that the enhancement in performance is mainly governed by the increase in powder content and the associated improvement in particle packing density, while the intrinsic effect of limestone powder plays a secondary role under the present experimental conditions. Therefore, although the current results demonstrate clear performance trends, the independent contribution of limestone powder cannot be fully quantified without a decoupled experimental design.

Overall, the mechanical performance of the concrete is primarily governed by the combined effects of powder content, particle packing, and microstructural refinement, rather than any single factor. The consistent non-monotonic trends observed in both compressive and flexural strength across different curing ages further confirm that limestone powder plays a multifaceted role in the system, extending beyond that of a simple inert filler. Although the variables in this study are inherently coupled, the observed trends provide meaningful insights into the underlying mechanisms. Future work should adopt decoupled experimental designs to isolate the individual contributions of powder content, particle size distribution, and packing characteristics.

#### 3.1.3. Coupled Influence of Replacement Rate and Total Powder Content

The coupled analysis demonstrated that the performance evolution could not be interpreted solely from limestone powder replacement rate. Since the increase in replacement level was accompanied by an increase in total powder content, the observed responses reflected their combined influence.

The results indicated that intermediate powder contents were beneficial for performance development, whereas excessive powder levels resulted in deterioration. Therefore, total powder content should be considered together with replacement ratio during mixture design. Future investigations should adopt decoupled experimental designs in which total powder content is independently controlled, thereby enabling rigorous quantification of the intrinsic contribution of limestone powder.

### 3.2. Durability Analysis

At 28 days of curing, the effect of limestone powder replacement on the chloride ion permeability of concrete with different water–binder ratios is presented in Figure 9. As in the case of mechanical properties, the increase in replacement rate is accompanied by a corresponding increase in total powder content, which necessitates a coupled interpretation of the results.

The chloride migration coefficient initially decreased and then increased with increasing replacement level. The optimum durability performance was obtained at intermediate replacement levels. The observed variation may be related to changes in internal transport characteristics induced by the coupled influence of replacement rate and powder content. Previous studies also reported that limestone powder may affect chloride transport behavior through changes in pore characteristics and chloride binding capacity [21].

It should be noted that the mixtures in this study incorporate supplementary cementitious materials (SCMs), including slag powder and fly ash, both of which exhibit pozzolanic or latent hydraulic activity. These materials may influence the hydration process and microstructural development of the concrete system. However, within each water–binder ratio group, the contents of slag and fly ash were kept constant. Therefore, the variations observed in mechanical properties and durability within each group can be primarily attributed to changes in the limestone powder replacement level. Nevertheless, potential interactions between limestone powder and the SCMs may contribute to the observed behavior. As a result, the experimental findings should be interpreted as the combined effect of multiple components within the system, rather than the influence of a single material.

### 3.3. Analysis of Mix Design for Limestone Powder Concrete

#### 3.3.1. Response Surface Method

It should be clarified that the initial mix proportions were determined based on standard design procedures (Section 2.3), while the response surface methodology was subsequently employed to analyze the experimental results and identify the optimal combination of variables. To determine the optimal mix proportion for limestone powder cement concrete, Design Expert software 13 was used with central composite design (CCD) for experimental design, establishing a standard CCD model with two factors and levels, using compressive strength, flexural strength, and chloride ion permeability resistance as responses, and developing regression equations to determine the optimal water–binder ratio and limestone powder replacement rate. Based on the aforementioned analysis, medium replacement rates showed the best performance; therefore, limestone powder replacement rates of 40%, 60%, and 80% were selected, with water–binder ratios of 0.38, 0.43, and 0.48. Accordingly, Table 6 shows the types and levels of factors for the response surface experiments. The response surface experimental results are presented in Table 7. The statistical significance of the developed regression models was evaluated by ANOVA. In addition to *p*-values and F-values, experimental variability was quantified using repeated measurements and confidence interval analysis to improve the reliability assessment of the developed models.

#### 3.3.2. Establishment of Regression Equations

Testing results are shown in Table 7. Based on the response surface method, multiple regression fitting was performed on the experimental data to obtain quadratic polynomial regression equations for compressive strength y_1_, flexural strength y_2_, and RCM value y_3_.

(3) $$y_{1}=299.09233-1114.26667x_{1}+1.045x_{2}+2.25x_{1}x_{2}+910x_{1}^{2}-0.015813x_{2}^{2}$$

(4) $$y_{2}=-0.455719+63.28772x_{1}-0.044509x_{2}+0.2x_{1}x_{2}-104.21053x_{1}^{2}-0.000526x_{2}^{2}$$

(5) $$y_{3}=26.23025-99.04895x_{1}-0.0989x_{2}+0.3325x_{1}x_{2}+107.78947x_{1}^{2}-0.000364x_{2}^{2}$$

Analysis of variance (ANOVA) was performed via Design Expert software 13 to validate the feasibility of the established model and evaluate the significance of both the model and its individual coefficients. Generally, the significance criteria are as follows: *p* < 0.01 indicates high significance; 0.01 ≤ *p* ≤ 0.05 indicates significance; and *p* > 0.05 indicates non-significance. The significance test results of the regression equations are shown in Table 8.

As shown in Table 8, the quadratic polynomial regression models for both 28-day compressive strength and chloride migration coefficient (RCM) exhibited *p*-values below 0.01, demonstrating high statistical significance, whereas the model for 28-day flexural strength showed a *p*-value marginally above 0.01, still indicating satisfactory significance. For compressive strength and flexural strength, the water–binder ratio has a very significant influence, while the replacement rate has a relatively significant influence. For the RCM value, the water–binder ratio has a very significant influence, while the replacement rate has no significant influence.

#### 3.3.3. Analysis of Response Surface

**Figure 10:**
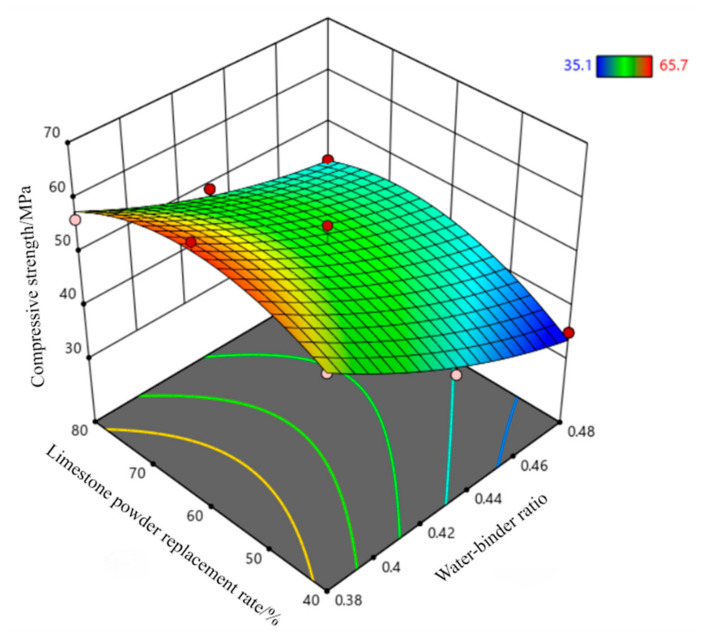
Response surface map of the compressive strength model.

Figure 10 shows the response surface plot of the interaction between two influencing factors on the compressive strength of limestone powder concrete. As shown in Figure 10, reducing the water–binder ratio generally leads to an increase in compressive strength, with the surface rising from blue to red. A low water–binder ratio makes the paste denser, reduces pores, and is likely to provide improved coating of aggregates of limestone powder and aggregates, thereby offsetting the increased water demand caused by limestone powder and promoting complete cement hydration. The effect of replacement rate on strength shows a unimodal trend: strength increases when rising from 40% to 60–70%, then decreases beyond that. At medium replacement rates, limestone powder optimizes gradation and enhances the interface, where water demand impact is not significant, but high replacement rates increase the total specific surface area of aggregates; insufficient paste leads to reduced coating and increased interface defects and may disrupt continuous gradation, raising void content. Considering the interaction, the optimal range is water–binder ratio 0.40–0.44 and replacement rate 60–70%, corresponding to the peak region of the surface.

**Figure 11:**
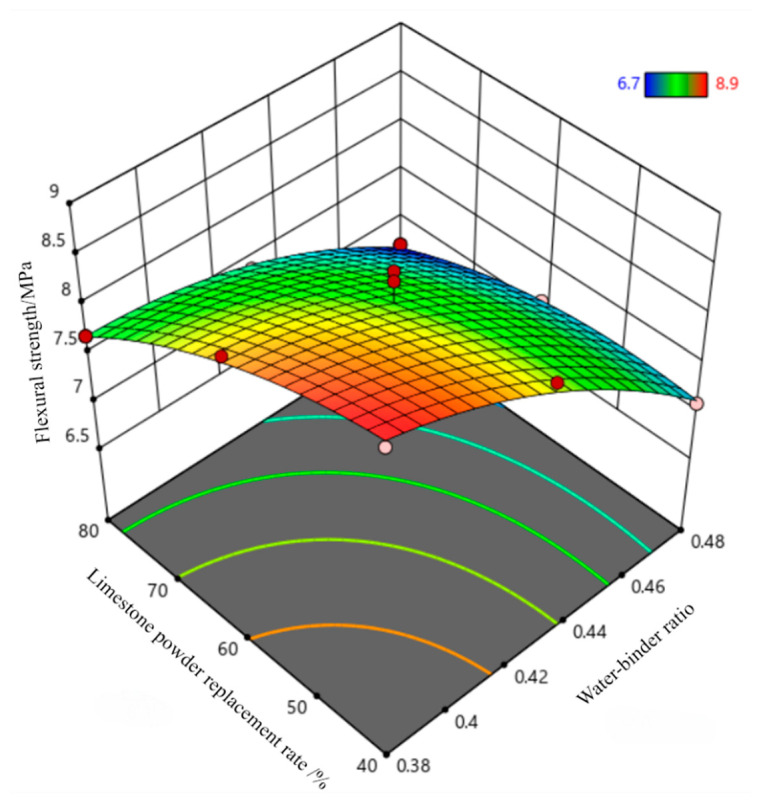
Response surface map of the flexural strength model.

Figure 11 shows the response surface plot of the interaction between two influencing factors on the flexural strength of limestone powder concrete. As shown in Figure 11, flexural strength increases with decreasing water–binder ratio and replacement rate, with its variation mainly controlled by both paste density and aggregate gradation. The contour lines show curved distribution, reflecting a nonlinear interaction between the two factors: At low replacement rates, the water–binder ratio has a more significant effect on flexural strength, as the natural sand skeleton remains stable and paste density plays a dominant role, whereas under high replacement rate conditions, the influence of water–binder ratio diminishes, as the aggregate skeleton effect is compromised and fine powder accumulation becomes the main factor limiting strength improvement. Overall, the combination of low water–binder ratio and replacement rate achieves both paste densification and skeleton stability, resulting in optimal flexural strength; conversely, high water–binder ratio combined with high replacement rate leads to porous paste and skeleton deterioration, producing the poorest strength. Specifically, within the 40–60% replacement range, limestone powder effectively fills aggregate gaps, optimizes gradation, and assists in enhancing density and strength, but when the replacement rate exceeds 60%, the sand skeleton effect is significantly weakened; issues such as fine powder accumulation and increased water demand become prominent, consequently reducing strength, and if combined with high water–binder ratio, strength will further decrease.

**Figure 12:**
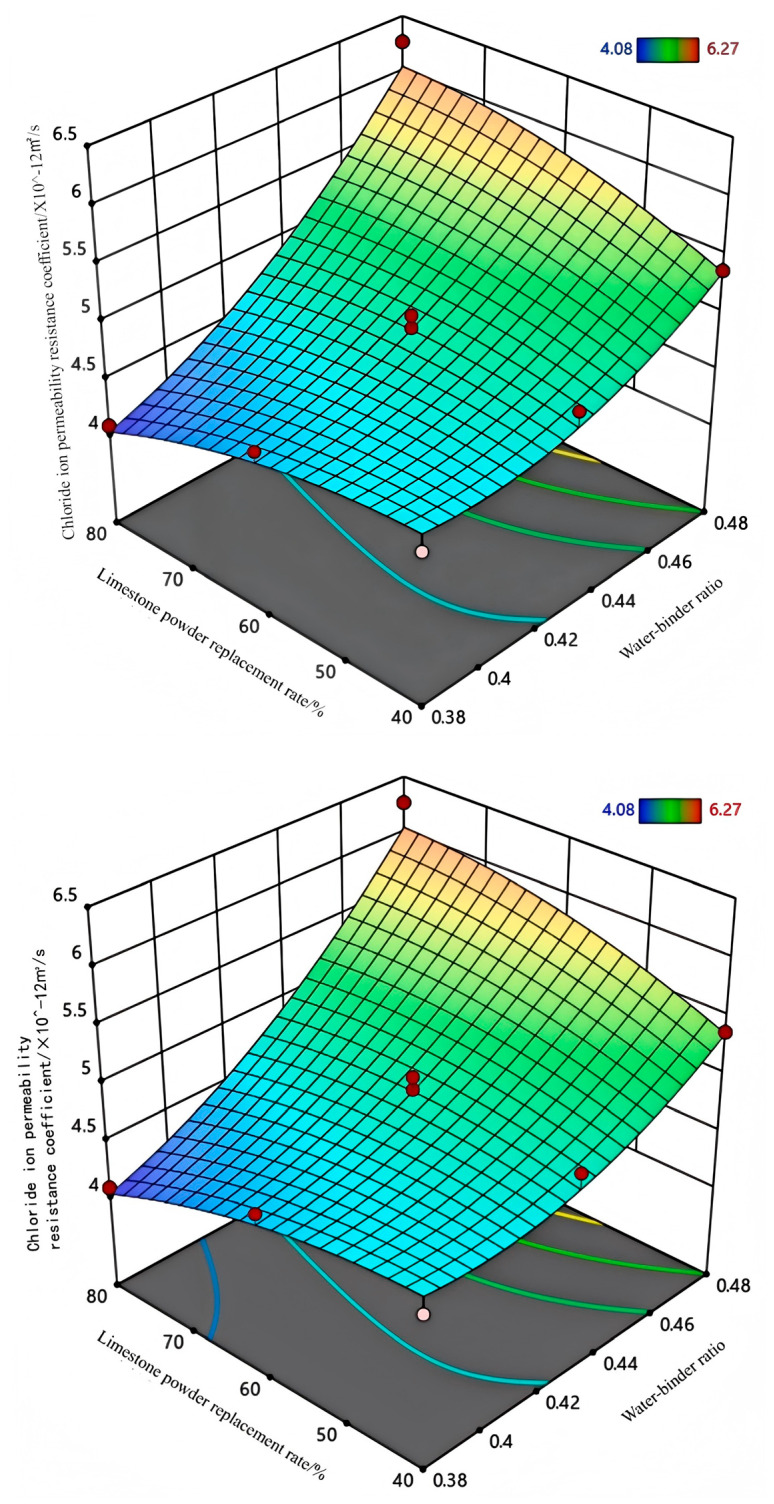
Response surface plot and contour plot of the chloride ion migration coefficient model.

Figure 12 shows the response surface plot of the interaction between two influencing factors on the durability of limestone powder concrete. As shown in the lower-left region of Figure 12 (low water–binder ratio is 0.38, and low replacement rate is 40%), the *Z*-axis value is the lowest, indicating optimal durability; in the upper-right region (high water–binder ratio is 0.48, and high replacement rate), the *Z*-axis value is the highest, indicating the poorest durability; the slope of the intermediate transition surface indicates that the water–binder ratio has a more significant influence, but the coupling effect of the replacement rate cannot be ignored. At low water–binder ratios, cement particles are in close contact, ion diffusion distance during hydration is short, and C-S-H gel formation is more sufficient and denser, with extremely low proportions of capillary and connected pores (chloride ions find it difficult to find continuous diffusion paths). At high water–binder ratios, cement particles are dispersed, hydration products are sparse, and capillary and connected pores remain abundant (porosity increases, and pore connectivity is strong), allowing chloride ions to migrate rapidly along the pores. At low replacement rates, fine mineral powder particles fill the gaps between sand particles, achieving tight packing, reducing macroscopic pores and connectivity, further hindering chloride ion migration (filling effect dominates). At high replacement rates, the skeletal support of sand is weakened, and the large specific surface area of mineral powder leads to a sharp increase in water demand (if water–binder ratio remains unchanged, the paste becomes thinner, and porosity increases), or fine powder accumulation forms “loose layers”, increasing chloride ion migration channels. The results of the study suggest that it is recommended to employ low water-to-binder ratios of less than or equal to 0.43, combined with moderate mineral powder substitution of less than or equal to 60%, to achieve a balance among durability, strength, and workability. In dry, low-chloride environments, the water–binder ratio and replacement rate can be appropriately increased, sacrificing some durability for cost advantages. Previous studies have reported that limestone powder may influence moisture transport, chloride ion transport, and permeability-related performance of concrete depending on replacement level and mixture design [31].

In summary, the theoretically optimal combination includes 0.38 water–binder ratio and 40% replacement rate, which achieves the highest strength level (without difference from 60%, significantly better than that of 80% or 0% replacement ratios), and shows no difference in RCM value from the optimal group (at 0% replacement ratio) for durability, significantly outperforming the degraded groups with 80% or 0% replacement ratios. However, the mixture with a water–binder ratio of 0.43 exhibited several favorable characteristics. Compared with the mixture with a water–binder ratio of 0.38, a higher slump value (180 ± 20 mm versus 150 ± 10 mm) was obtained, indicating improved workability under the present experimental conditions. In addition, the lower cement content associated with the water–binder ratio of 0.43 may contribute to reduced material consumption. Previous studies have reported that mixtures with improved workability and reduced binder content may exhibit advantages regarding construction performance and temperature control under specific engineering conditions [32]. Nevertheless, pumping behavior, crack resistance, and field applicability were not evaluated in the present study. Therefore, the present results should be regarded as engineering references for mixture design rather than direct engineering recommendations. Additionally, costs are reduced, improving project profitability; therefore, the combination of 0.43 water–binder ratio and 60% replacement rate can be selected as the recommended application combination.

### 3.4. Strain Cloud Diagrams Based on DIC Technology

**Figure 13:**
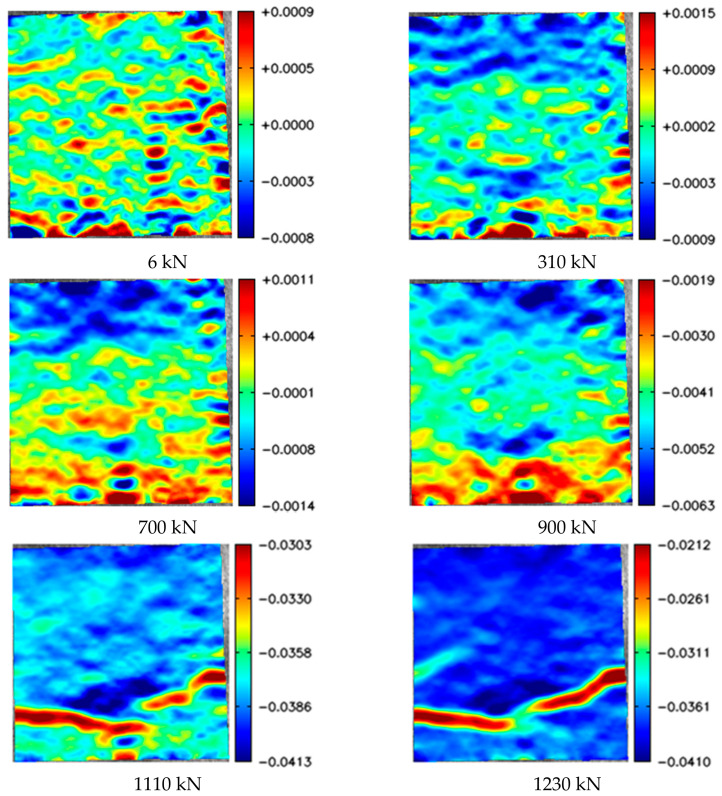
Strain cloud map of Group 0.38.

**Figure 14:**
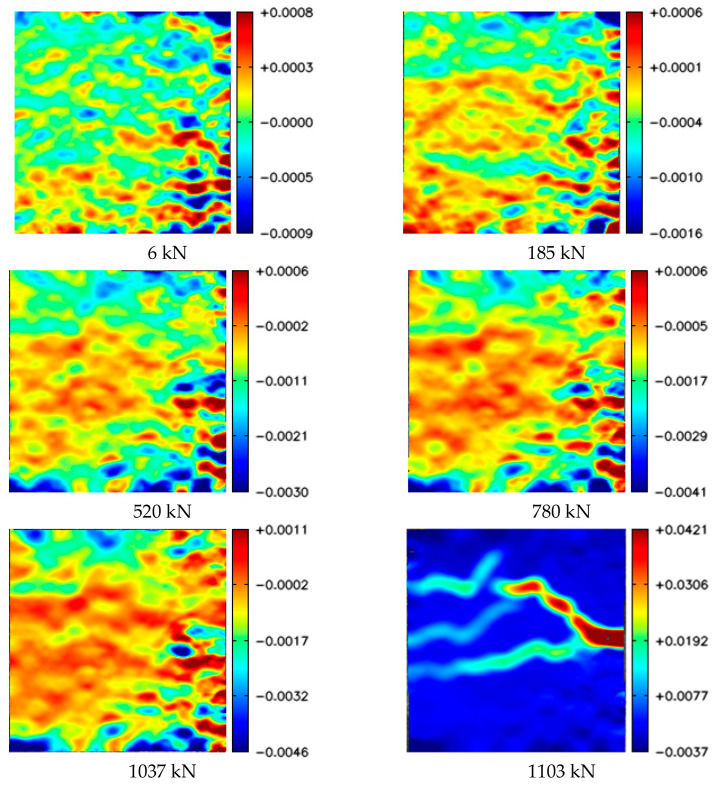
Strain cloud map of Group 0.43.

**Figure 15:**
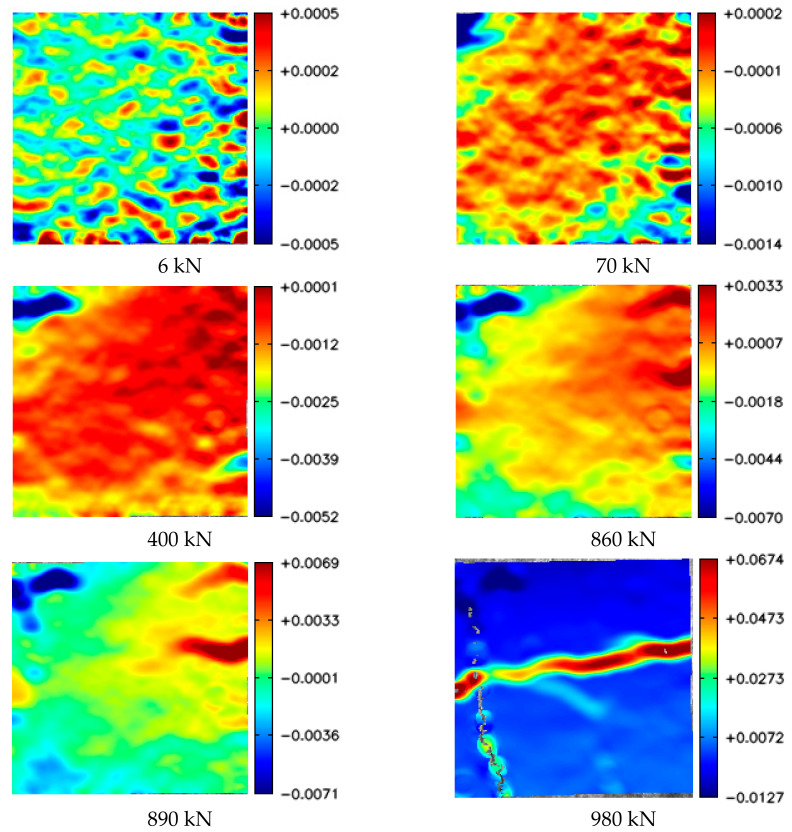
Strain cloud map of Group 0.48.

During the test loading process, DIC was used to record the stress–strain state on the concrete surface, and the strain cloud maps from DIC were used to depict the location and development trend of concrete cracks. Figure 13, Figure 14 and Figure 15 respectively show the variations in strain cloud diagrams for the 0.38, 0.43, and 0.48 groups under compressive testing.

The DIC results demonstrated the progressive transition from distributed deformation to localized fracture. Although all specimens exhibited similar failure evolution, different localization intensities were observed. Figure 14 presents the strongest localization behavior, whereas Figure 15 exhibits relatively broader deformation distribution.

The quantitative assessment of the DIC results is shown in Table 9. Quantitative assessment of the DIC results indicated that strain localization evolved progressively during loading. Figure 13, Figure 14 and Figure 15 all exhibit the transition from distributed deformation to localized fracture. However, the localization intensity differed among the specimens. Figure 14 shows the strongest localization behavior with penetrating crack bands and the highest strain localization index (SLI = 3.0), whereas Figure 13 exhibits delayed localization development. Figure 15 presents a relatively broader strain distribution at intermediate stages, indicating more diffuse deformation evolution.

### 3.5. Integrated Discussion

The experimental results consistently indicated that the performance evolution could not be interpreted solely from limestone powder replacement level. Since the increase in replacement rate was accompanied by increased powder content, the observed responses reflected their combined influence.

Intermediate replacement levels generally produced favorable strength and chloride resistance performance, whereas excessive powder content resulted in deterioration. The DIC analysis further confirmed differences in deformation localization behavior among mixtures.

Therefore, replacement level and total powder content should be considered simultaneously during mixture design.

## 4. Conclusions and Limitations

### 4.1. Study Limitations

(1) The present study contains an inherent limitation associated with the experimental design. Since limestone powder was introduced by replacing natural sand, the increase in replacement rate inevitably resulted in a simultaneous increase in total powder content. Consequently, the independent effect of limestone powder could not be isolated from that of powder content. Therefore, the findings should be interpreted as coupled responses. Future studies should maintain constant powder content while varying limestone powder dosage to rigorously evaluate intrinsic material effects.
(2) The present study mainly focused on macroscopic performance evaluation. Direct characterization methods for investigating interfacial transition zone evolution, hydration products, pore structure, and transport mechanisms were not performed. Therefore, interpretations concerning ITZ improvement, hydration enhancement, particle packing modification, and chloride transport behavior should be regarded as possible explanations inferred from macroscopic responses rather than experimentally verified mechanisms. Future studies incorporating XRD, MIP, TG/DTG, and porosity characterization are required for rigorous validation.

### 4.2. Conclusions

Compared with previous studies mainly focusing on limestone powder as a cementitious replacement, this study emphasized its application as a fine aggregate substitute and highlighted the coupled effect between replacement rate and total powder content. The integration of RSM optimization and DIC characterization further expanded the understanding of performance evolution and crack development mechanisms in limestone powder concrete systems. Based on the experimental results and analysis, the following conclusions can be drawn:

(1) The mechanical properties exhibited a typical increasing–decreasing trend with increasing limestone powder replacement level. Intermediate replacement levels generally provided favorable performance, whereas excessive replacement caused deterioration. The optimum replacement range was mainly concentrated at intermediate levels.
(2) The chloride ion migration coefficient also showed a decreasing–increasing tendency with increasing replacement level. Improved chloride resistance was generally obtained at intermediate replacement rates. However, the transport mechanisms were not directly investigated and therefore remain to be further verified through microstructural characterization.
(3) Using the DIC analysis method revealed progressive deformation evolution from distributed strain to localized fracture. Quantitative assessment indicated different localization intensities among specimens, and the deformation process gradually evolved toward concentrated failure at later loading stages.
(4) The results demonstrated that the performance evolution of limestone powder concrete could not be interpreted solely from replacement level. Since increasing replacement simultaneously increased total powder content, the observed responses reflected their combined influence rather than the isolated effect of limestone powder. Therefore, replacement level and total powder content should be jointly considered during mixture design.
(5) The optimization results indicated that mixtures with intermediate replacement levels generally exhibited balanced performance regarding strength, chloride resistance, and workability. The optimized mixtures may provide engineering references for the design and application of limestone powder concrete.
(6) The present study established an integrated evaluation framework considering mechanical performance, chloride resistance, deformation evolution, and optimization analysis. The findings provide theoretical support and practical guidance for the engineering application and proportion design of limestone powder concrete.

## Figures and Tables

**Table 1:** Basic indicators of limestone powder.

Apparent Density/(kg/m^3^)	Through Sieve Hole Percentage/%		
	2.36 mm	0.6 mm	0.15 mm
2680	14.2	57.2	92.6

**Table 2:** Chemical composition of limestone powder (wt%).

Component	CaO	SiO_2_	Fe_2_O_3_	MgO	Al_2_O_3_	SO_3_	K_2_O
Content (%)	58.26	0.79	0.12	0.47	0.23	0.01	0.09

**Table 3:** Design grading table.

Aggregate Specification		Proportion (%)	Mass Percentage (%) Through the Following Sieve Hole Dimensions (mm)							
			37.5	31.5	26.5	19	16	9.5	4.75	2.36
Raw material gradation	16–31.5 mm crushed stone	100	100	98.29	62.06	3.58	1.15	0.43	0.43	0.43
	10–20 mm crushed stone	100	100	100	98.57	62.08	27.65	0.46	0.32	0.32
	5–10 mm crushed stone	100	100	100	100	100	100	89.30	10.70	0.78
Gradation of various aggregates in mixture	16–31.5 mm crushed stone	13	13.00	12.78	7.93	0.28	0.00	0.00	0.00	0.00
	10–20 mm crushed stone	70	70.00	70.00	69.00	42.83	11.84	0.05	0.00	0.00
	5–10 mm crushed stone	17	17.00	17.00	17.00	17.00	17.00	15.18	1.62	0.01
Synthetic gradation			100.00	99.78	93.93	60.12	28.85	15.24	1.62	0.01
Median design gradation			100.00	97.50	/	70.00	/	20.00	5.00	2.50
Design gradation range			100.00	95~100	/	55~85	/	10~30	0~10	0~5

**Table 4:** Test design and material scale.

Water–Binder Ratio	Limestone Powder Replacement Rate/%	Material Dosage/(kg/m^3^)							
		Water	Cement	Slag Powder	Fly Ash	Crushed Stone	Sand	Stone Chips	Chemical Admixture
0.38	0	165	262	103	69	1028	774.0	0.0	8.68
	20						619.2	154.8	
	40						464.4	309.6	
	60						309.6	464.4	
	80						154.8	619.2	
	100						0.0	774.0	
0.43	0		232	91	61	1037	814.0	0.0	7.68
	20						651.2	162.8	
	40						488.4	325.6	
	60						325.6	488.4	
	80						162.8	651.2	
	100						0.0	814.0	
0.48	0		208	82	54	1040	851.0	0.0	6.88
	20						680.8	170.2	
	40						510.6	340.4	
	60						340.4	510.6	
	80						170.2	680.8	
	100						0.0	851.0	

**Table 5:** Powder content.

Water–Binder Ratio	Limestone Powder Replacement Rate/%	Powder (kg/m^3^)
0.38	0	434
	20	588.8
	40	743.6
	60	898.4
	80	1053.2
	100	1208
0.43	0	384
	20	546.8
	40	709.6
	60	872.4
	80	1035.2
	100	1198
0.48	0	344
	20	514.2
	40	684.4
	60	854.6
	80	1024.8
	100	1195

**Table 6:** Factors and levels of response surface method.

Level	Factor	
	A: Water–Binder Ratio	B: Limestone Powder Replacement Rate/%
1	0.38	40
2	0.43	60
3	0.48	80

**Table 7:** Design and results of response surface experiment.

Water–Binder Ratio	Replacement Rate/%	Compressive Strength/MPa	Flexural Strength/MPa	Chloride Ion Permeability Resistance Coefficient
0.38	40	57.7	8.9	4.52
	60	65.7	8.6	4.59
	80	56.4	7.7	4.08
0.43	40	42.0	8.4	4.89
	60	51.6	8.4	5.03
	60	51.8	7.5	4.90
	60	55.4	8.3	4.92
	60	50.4	/	/
	80	49.4	7.4	4.49
0.48	40	35.1	7.1	5.38
	60	42.9	7.1	5.62
	80	42.8	6.7	6.27

**Table 8:** Analysis of variance of test results.

Level	Compressive Strength	Flexural Strength	RCM Value
	*p*	*p*	*p*
Model	0.0004	0.0141	0.0062
X_1_: Water–binder ratio	<0.0001	0.0027	0.0007
X_2_: Replacement rate	0.0499	0.0208	0.9310
X_1_^2^	0.1575	0.2499	0.1141
X_2_^2^	0.0041	0.3412	0.3491
X_1_X_2_	0.0982	0.2649	0.0313
Undrafted item	0.6051	0.9916	0.0579

**Table 9:** Quantitative characterization of DIC strain localization.

Figure	Stage	Strain Localization Level	SLI	Failure Mode
Figure 13	Initial–middle stage	Weak–moderate	1.5	Distributed deformation
Figure 13	Peak stage	Strong	2.4	Single diagonal localization band
Figure 14	Initial–middle stage	Moderate	1.8	Progressive localization
Figure 14	Peak stage	Very strong	3.0	Penetrating crack band
Figure 15	Initial–middle stage	Moderate–strong	2.2	Extended strain concentration
Figure 15	Peak stage	Strong	2.6	Localized fracture

## Data Availability

The original contributions presented in this study are included in the article/Supplementary Materials. Further inquiries can be directed to the corresponding author.

## Supplementary Materials

The following supporting information can be downloaded at https://www.mdpi.com/article/10.3390/ma19112348/s1.
