# Supplementary material for: Mix Design and Performance Analysis of Concrete with Limestone Powder Admixture
Source: Materials (Basel). 2026 Jun 2;19(11):2348. doi: 10.3390/ma19112348 (PMC13258419; doi:10.3390/ma19112348)
Supplement: Supplementary file 1 [file materials-19-02348-s001.zip › materials-4320003-supplementary.pdf]

Supplementary Materials

Table S1 Statistical summary of compressive strength results

W/B	Replacement (%)	Mean (MPa)	SD	95% CI
0.38	0	42.4	1.11	42.4±2.76
0.38	20	48.1	1.45	48.1±3.60
0.38	40	57.7	1.11	57.7±2.76
0.38	60	65.7	1.27	65.7±3.15
0.38	80	56.4	0.44	56.4±1.09
0.38	100	45.3	0.38	45.3±0.94
0.43	0	42.9	0.69	42.9±1.71
0.43	20	44.6	0.37	44.6±0.92
0.43	40	42.0	1.22	42.0±3.03
0.43	60	52.0	1.24	52.0±3.08
0.43	80	49.4	1.50	49.4±3.73
0.43	100	43.5	0.45	43.5±1.12
0.48	0	37.1	0.49	37.1±1.22
0.48	20	32.1	0.59	32.1±1.47
0.48	40	35.1	0.18	35.1±0.45
0.48	60	42.9	0.57	42.9±1.42
0.48	80	42.8	0.99	42.8±2.46
0.48	100	37.4	0.36	37.4±0.89

Table S2 Statistical summary of flexural strength results

W/B	Replacement (%)	Mean (MPa)	SD	95% CI
0.38	0	7.4	0.20	7.4±0.50
0.38	20	6.9	0.10	6.9±0.25
0.38	40	8.9	0.08	8.9±0.20
0.38	60	8.6	0.17	8.6±0.42
0.38	80	7.7	0.06	7.7±0.15
0.38	100	7.9	0.24	7.9±0.60
0.43	0	7.0	0.16	7.0±0.40
0.43	20	6.6	0.19	6.6±0.47
0.43	40	8.4	0.10	8.4±0.25
0.43	60	8.1	0.18	8.1±0.45
0.43	80	7.4	0.15	7.4±0.37
0.43	100	7.5	0.12	7.5±0.30
0.48	0	6.3	0.16	6.3±0.40
0.48	20	6.2	0.19	6.2±0.47
0.48	40	7.1	0.17	7.1±0.42
0.48	60	7.1	0.26	7.1±0.65
0.48	80	6.7	0.15	6.7±0.37
0.48	100	6.6	0.18	6.6±0.45

Table S3 Statistical summary of chloride migration coefficient results

W/B	Replacement (%)	Mean	SD	95% CI
0.38	0	4.87	0.14	4.87±0.35
0.38	20	4.63	0.16	4.63±0.40
0.38	40	4.52	0.09	4.52±0.22
0.38	60	4.59	0.15	4.59±0.37
0.38	80	4.08	0.14	4.08±0.35
0.38	100	6.47	0.14	6.47±0.35
0.43	0	4.91	0.17	4.91±0.42
0.43	20	4.19	0.12	4.19±0.30
0.43	40	4.89	0.20	4.89±0.50
0.43	60	5.03	0.17	5.03±0.42
0.43	80	4.49	0.18	4.49±0.45
0.43	100	6.10	0.17	6.10±0.42
0.48	0	5.26	0.18	5.26±0.45
0.48	20	5.83	0.12	5.83±0.30
0.48	40	5.38	0.10	5.38±0.25
0.48	60	5.62	0.14	5.62±0.35
0.48	80	6.27	0.18	6.27±0.45
0.48	100	6.34	0.19	6.34±0.47
